# Cognitive Flexibility in ASD; Task Switching with Emotional Faces

**DOI:** 10.1007/s10803-012-1512-1

**Published:** 2012-03-29

**Authors:** Marieke de Vries, Hilde M. Geurts

**Affiliations:** 1 Brain and Cognition, Department of Psychology, University of Amsterdam, Weesperplein 4, 1018 XA Amsterdam, The Netherlands; 2 Autism Outpatient Clinic, Dr. Leo Kannerhuis, Amsterdam, The Netherlands; 3 d’Arc, Dutch Autism and ADHD Research Center, Amsterdam, The Netherlands

**Keywords:** ASD, Task switching, Cognitive flexibility, Emotion, Executive functioning

## Abstract

Children with autism spectrum disorders (ASDs) show daily cognitive flexibility deficits, but laboratory data are unconvincing. The current study aimed to bridge this gap. Thirty-one children with ASD (8–12 years) and 31 age- and IQ-matched typically developing children performed a gender emotion switch task. Unannounced switches and complex stimuli (emotional faces) improved ecological validity; minimal working memory-load prevented bias in the findings. Overall performance did not differ between groups, but in a part of the ASD group performance was slow and inaccurate. Moreover, within the ASD group switching from emotion to gender trials was slower than vice versa. Children with ASD do not show difficulties on an ecological valid switch task, but have difficulty disengaging from an emotional task set.

## Introduction

Autism spectrum disorders (ASDs), including autism, Asperger syndrome and pervasive developmental disorder not otherwise specified (PDD-NOS), are neurobiological developmental disorders in which restricted, repetitive behaviors and interests, and social and communicational problems predominate (American Psychiatric Association [APA] 2000). Children with ASD have deficits in executive functioning (Russell 1997), especially cognitive flexibility. Cognitive flexibility is the ability to switch rapidly between multiple tasks (Monsell 2003). Individuals with ASD have trouble adapting to variable demands of the environment (Kenworthy et al. 2010), show rigid behavior, hold on to previous behavior patterns, and show difficulty in adapting to changing plans or alterations of their routine in daily life. This restricted and repetitive behavior seems to be closely related to, or even an expression of impairment of, cognitive flexibility (Yerys et al. 2009). Although cognitive flexibility deficits in everyday life seem evident, the empirical laboratory data is not convincing (Geurts et al. 2009b). It seems that individuals with ASD perform better on computerized than on face to face administered tasks (Kenworthy et al. 2008), but the data is still inconclusive on both types of tasks (Van Eylen et al. 2011). In a recent review it was reported that the findings regarding cognitive flexibility in ASD are not merely inconsistent, but sometimes even contradictory (Geurts et al. 2009b). This inconsistency might be a result of the heterogeneity of the cognitive profiles in ASD, the high levels of comorbidity, and the overlap of different executive functions (Kenworthy et al. 2008). It is hard to find an accurate way to measure the construct of cognitive flexibility in the laboratory setting, and most tasks seem to lack ecological validity (Kenworthy et al. 2008). In the current study we try to bridge the gap between cognitive flexibility deficits as seen in everyday life and as measured in the laboratory setting.

In short, we used three kinds of tasks to measure cognitive flexibility in the research-setting; (1) traditional clinical neuropsychological measures; such as the Wisconsin Card Sorting Task (WCST), Trail Making Test (TMT), or Dellis-Kaplan executive function system (D-KEFS) color-word task; (2) hybrid neuropsychological/experimental measures, such as the intra-dimensional/extra-dimensional (ID/ED) set shift task (Cambridge Cognition 1996); and (3) experimental task-switch paradigms, for instance, switch tasks (Geurts et al. 2009b; Monsell 2003). Children with ASD show deficits on the WCST, and the D-KEFS color word task (Van Eylen et al. 2011). However, these are no a pure cognitive flexibility measurements (Geurts et al. 2009b, Ozonoff 1995), as they measure various cognitive functions, like working memory (WM), learning from feedback, and noticing changes in strategy. Switches in these tasks often occur in both an unpredictable and an unannounced manner. This makes it hard to disentangle what causes the failure on these tasks. Moreover, performance on these tasks is influenced by developmental level, which could also be accountable for the results (Happé et al. 2006). On the ID/ED shift task, results are also varying (Yerys et al. 2009). In this task it is necessary to shift within a single dimension (ID), from one dimension to another (ED), and to shift reversed (applying the same rule to an alternate exemplar; Cambridge Cognition 1996). Children with ASD show deficits in the ED reversal shifts, but do not in other shifts (Yerys et al. 2009, but see Happé et al. 2006). Apparently, when more stimulus-features have to be processed and more complex reasoning is necessary, children with ASD show cognitive flexibility deficits. Sustained attention also influences task performance (Geurts et al. 2009b), and because children with ASD experience attention deficits (Patten and Watson 2011), it might be that cognitive flexibility alone is not accountable for failure on these hybrid neuropsychological/experimental measures. In sum, it seems that traditional clinical neuropsychological measures, and hybrid neuropsychological/experimental measures, are not pure cognitive flexibility measurements; the findings on these measures seem to be influenced by many other variables.

The experimental task switch paradigm, for instance, switch task, is a relatively pure measurement of cognitive flexibility. Stimuli have to be sorted on two (or more) simple rules, for instance, sorting on color or sorting on form. After a number of consecutive trials performing one task (repeat trials), the other task has to be performed (switch trial). Performance is known to be slower and less accurate on switch trials than on repeat trials. The measures of performance usually are error rate (percentage of trials answered incorrectly), switch cost in reaction time (reaction time on switch trials minus reaction time on repeat trials) and switch cost in number of errors (error rate on switch trials minus error rate on repeat trials; Monsell 2003). Several studies that used switch tasks to compare children with ASD with typically developing (TD) children, revealed inconsistent results (Geurts et al. 2009b).

A possible explanation is that children with ASD simply do not have cognitive flexibility deficits, but given the prominent cognitive flexibility deficits exhibited in daily life, and the link between repetitive behavior and cognitive flexibility (Lopez et al. 2005; Yerys et al. 2009, but see Landa and Goldberg 2005), this conclusion seems too rigorous. The current study tries to overcome the conflicting findings by taking into account several task properties. Up to now the studied switch tasks roughly differ on three dimensions; WM-load, predictability, and the used stimuli. These differences influence both performance and ecological validity, and will be discussed in the next paragraphs.

Firstly, WM-load influences performance. In daily life, various tasks are influenced by both cognitive flexibility and WM, for instance, interacting with people in various situations requires both flexibility in interpreting the situation, and flexibility in remembering and processing information. Individuals with ASD show WM deficits (Alloway et al. 2009; Barnard et al. 2008, but see Happé et al. 2006). Both verbal, and spatial WM seems deficient (Kenworthy et al. 2008, Willcutt et al. 2008, but see Geurts et al. 2004), but some argue that the deficits in visual-spatial WM are the most prominent (Williams et al. 2005, 2006). Switch tasks rely on WM because arbitrary rules need to be memorized. The amount of WM-load varies (Dichter et al. 2010; Stoet and López 2010); the cue predicting a task switch can be available during each trial (Schmitz et al. 2006), at the beginning of a task run (Poljac et al. 2010; Shafritz et al. 2008), or at the beginning of the whole task (Maes et al. 2010). Hence, the poor WM in ASD is likely to influence switch task performance and might partly explain the inconsistent findings.

The joint influence of WM and cognitive flexibility as seen in everyday life is confirmed in the research setting (Stoet and López 2010). On switch tasks with minimal WM demand, children with ASD do not show difficulties (Schmitz et al. 2006; Stoet and López 2010), whereas difficulties are reported when the WM demand is higher (Maes et al. 2010; Shafritz et al. 2008; Stoet and López 2010, but see Poljac et al. 2010). In fact, performance on switch tasks seems to be more influenced by WM demand in children with ASD than in TD children (Dichter et al. 2010; Stoet and López 2010). Children with ASD are less accurate than TD children, and show larger switch costs when performing a switch task with high memory demand. However, on a similar task with low memory demand, both groups perform equal (Stoet and López 2010). Hence, WM capacity influences switch task performance (Alloway et al. 2009; Karbach and Kray 2009; Williams et al. 2005), especially in ASD (Stoet and López 2010). This partly explains the inconsistent findings, as the varying WM demand in switch tasks is often not controlled for. In the current study, a task cue is always present, so that WM capacity cannot influence performance.

Secondly, switches can occur in a predictable (Shafritz et al. 2008), or an unpredictable manner (Maes et al. 2010). Put differently, switches can occur after every other trial, and can be preceded by a switch cue (i.e., predictable switches) or occur after a varying inconsistent numbers of trials, and occur completely unannounced (i.e., unpredictable switches). Children with ASD do not show deficits on switch tasks with predictable switches (Stahl and Pry 2002; Whitehouse et al. 2006), but do show deficits when switches occur unpredictably (Maes et al. 2010; Stoet and López 2010; Yerys et al. 2009, but see Schmitz et al. 2006). In the current study a switch task with unpredictable switches is used to measure cognitive flexibility, especially since this increases ecological validity, as in everyday life the need for behavioral adaptation is normally not preceded by a warning.

Thirdly, most studies use simple geometrical figures as stimuli (Maes et al. 2010; Poljac et al. 2010; Schmitz et al. 2006; Shafritz et al. 2008; Stahl and Pry 2002; Stoet and López 2010), minimizing WM-load, and mental processing, but also reducing ecological validity. These tasks seem unable to discriminate ASD from TD individuals (Poljac et al. 2010; Schmitz et al. 2006; Stahl and Pry 2002; Stoet and López 2010). In everyday life, cognitive flexibility is needed while processing complex stimuli and more specifically, while participating in social interaction, as people tend to act differently in various situations. In the current study, ecological validity has been improved by administering a switch task with relatively complex and socially relevant stimuli in the form of male and female faces with different facial expressions. Children with ASD process emotions in a different way than TD individuals (Santos et al. 2008). An enhanced focus on irrelevant details leads to a reduced ability to recognize emotions (Begeer et al. 2008), and reacting to other people’s emotions appears to be difficult (Golan et al. 2008). Emotion processing also influences rigidity and deficits in social interactions in ASD (APA 2000), like adapting behavior and perspective, and inhibiting inappropriate behavior (Causton-Theoharis et al. 2009). This specific way of processing emotions—both in the laboratory setting (Santos et al. 2008), and in everyday life (Begeer et al. 2008)—is likely to influence switch task performance in the current task. To prevent that task performance would be influenced by emotion recognizing problems per se, we only used basic emotions. In the laboratory setting individuals with ASD do recognize basic emotions (Balconi et al. 2012; Boggs and Gross 2010), and are equally able as TD children to differentiate between happy and angry faces (Geurts et al. 2009a, Santos et al. 2008). Children with ASD also seem very well able to categorize faces by emotion or gender (Harms et al. 2010; Santos et al. 2008). In short, the distinct way children with ASD process emotional faces is thought to influence both everyday life behavior and performance on the current task. Hence, compared to most switch tasks, using simple geometric forms as stimuli, the ecological validity is improved. In daily life, interpreting emotions and gender is necessary in social interaction, but sorting on color or form is hardly ever needed.

In sum, in the current study we tried to bridge the gap between daily life cognitive flexibility deficits, and laboratory setting cognitive flexibility measurements in ASD. We compared the performance of a clinical group of children with ASD and an age- and IQ-matched TD group on a gender emotion switch task. Participants sorted pictures of happy or angry looking male or female faces, based on gender or emotion, randomly switching between the two sorting rules. A standardized switch task with a constant present task cue was used as a relatively pure measurement of cognitive flexibility with no WM influence. Switches occurred unannounced, and stimuli consisted of faces to improve ecological validity. The faces showed basic emotions, as these are recognized well by children with ASD (Boggs and Gross 2010; Geurts et al. 2009a), ensuring that emotion recognition deficits would not influence the findings. The difference between individuals with ASD and TD in processing faces (Santos et al. 2008) and emotions (Balconi et al. 2012) was expected to influence cognitive flexibility in everyday life and on this task similarly.

Firstly, performance on the gender emotion switch task was expected to be worse in children with ASD than in TD children, due to the unannounced switches (Maes et al. 2010; Stoet and López 2010; Yerys et al. 2009), and the increased ecological validity. Secondly, we expected that children with ASD that performed relatively poorly on the gender emotion switch task, would have higher scores on the ‘stereotyped behavior’, and ‘fear of changes’ scales of the Children’s Social Behavior Questionnaire (CSBQ; Luteijn et al. 1998), and the ‘repetitive behavior’ scale of the Autism Diagnostic Interview Schedule-Revised (ADI-R: Lord et al. 1994). Thirdly, children with ASD were expected to have trouble to disengage attention from an emotional task set, resulting in worse performance on emotion to gender switch trials than vice versa.

The last hypothesis is based on the assumption that various emotions are processed differently (Harms et al. 2010; Johnson 2009). For example, angry faces are perceived as more threatening and consequently remembered better than happy faces, and are more resistant to modification (Willis et al. 2010). Emotional stimuli influence task performance in general (Johnson 2009), and probably differently in individuals with and without ASD, because of the dissimilar emotion processing (Santos et al. 2008). Individuals that score high on a trait anxiety scale, and a worrisome thoughts scale of a stress state questionnaire have trouble disengaging their attention from an emotional task set, when switching to a neutral task set (Johnson 2009). These individuals have less effective emotional attention control, and poorly regulated attentional deployment; a strategy to reduce emotional reactivity by shifting attention away from emotion-eliciting stimuli (Johnson 2009). This dysregulation in attentional deployment, or attentional inflexibility, has been linked to anxiety, depression, and ASD (Maes et al. 2010), and was expected to influence the performance of the children with ASD in the current study.

## Method

### Participants

Thirty-five children with ASD and 35 age-, IQ-, and gender-matched TD children participated in this study. Children with ASD were recruited through several mental health care clinics in the Netherlands, and all had a clinical diagnosis according to the DSM-IV criteria for an ASD such as autism, Asperger syndrome or PDD-NOS (American Psychiatric Association 2000). The children were all diagnosed by a multidisciplinary team specialized in the assessment of children with ASD. Moreover, in the current study only those children were included who scored above the Dutch cut-off for ASD (score of 65; Roeyers et al. 2011) on the Social Responsiveness Scale parent report (SRS; Constantino et al. 2003). In addition, to verify the diagnosis, also the ADI-R (Lord et al. 1994) was administered. Children reaching specified cut-offs in at least two of the three specified domains were included in the current study (Gray et al. 2008; Rutter et al. 2003). One child did not meet the cut-off score of 65 on the SRS (Charman et al. 2007), but as the score approached the cut-off, the ADI-R was still administered. All children reached the specified ADI-R cut-off scores, and were, therefore, included (see Table 1).

**Table 1:** Means (standard deviation) demographic and clinical scores ASD and TD

Measure	Group				Group comparison	
	ASD (*n* = 31)	Range	TD (*n* = 31)	Range	*T* value (60)	*p*
Gender (boys/girls)	25/6		25/6			
Age (years)	10.5 (1.4)	8.1–12.9	10.5 (1.1)	8.2–12.5	0.28	0.31
FSIQ	108.7 (20.0)	81–149	109.4 (19.8)	80–154	−0.13	0.88
SRS	105.0 (24.0)	61–149	25.4 (15.4)	5–58	15.6	<0.05
CSBQ	48.7 (11.5)	29–69	7.8 (7.3)	0–24	−16.4	<0.01
ADI-R						
Social interaction	20.0 (4.5)	10–27				
Communication	16.3 (3.4)	8–14				
Repetitive behavior	4.8 (2.4)	1–10				
Visible < 36 months	3.0 (1.0)	1–5				

*ASD* autism spectrum disorder, *TD* typically developing. The ASD group consisted of children with an clinical diagnosis of Autism (*n* = 9), Asperger’s syndrome (*n* = 10), and PDD-NOS (*n* = 12). For 24 children the cut-off was reached on all three scales of the ADI-R and for 7 children the cut-off was reached on the social and communication scale but not on the repetitive behavior scale. For all children the onset was before 3 years of age. *FSIQ* Full Scale Intelligence Quotient (Sattler 2001). *SRS* Social Responsiveness Scale (Constantino et al. 2003). *CSBQ* Children’s Social Behavior Questionnaire (Luteijn et al. 1998). *ADI-R* Autism Diagnostic Interview Schedule-Revised (Lord et al. 1994)

TD children were recruited via two primary schools in the Netherlands. Screening questionnaires were administered; children having a psychiatric or developmental disorder, taking psychotropic drugs, or scoring above the ASD cut-off on the SRS were excluded.

Further inclusion criteria for both groups were (1) between 8 and 12 years of age; (2) IQ scores >80; and (3) absence of seizure disorders. Three children in the ASD group had an estimated IQ-score below 80 and were excluded from participation as well as the age-, IQ-, and gender-matched children from the TD group. Based on the SRS score, one child of the TD group was excluded from participation, as well as the age-, IQ-, and gender-matched child from the autism group. Thirty-one children with ASD met the inclusion criteria and were included in the present study (25 boys and 6 girls). Thirty-one children from the TD group, matched on age, estimated IQ scores, and gender were included (for details see Table 1). Eleven of the ASD participants and none of the TD participants used psychotropic medication on a permanent basis.

### Measures

#### Gender/Emotion Switch Task

**Fig. 1:**
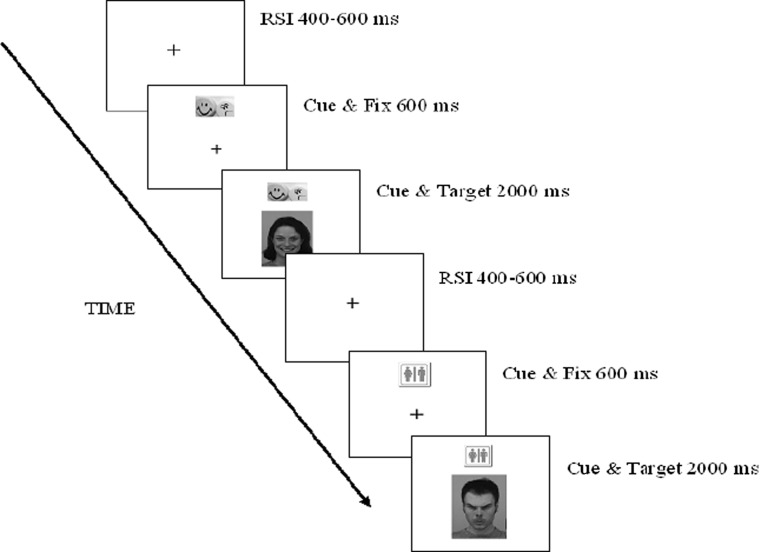
Schematic illustration of two sample trials from the gender-emotion switch task. *RSI* response stimulus interval. Time is in milliseconds

The gender-emotion switch task is an adaptation of the classical switch task (Rogers and Monsell, 1995; White and Shah 2006). Pictures of two male and two female faces, looking angry or happy, were displayed on the computer screen. Participants alternated between reporting the emotion or the gender of a face by pressing a left or right button on the keyboard. Participants were instructed which button referred to happy, angry, male, and female. A central fixation cross appeared (400–600 ms) on the screen during each trial, followed by the fixation cross together with the task cue (600 ms) consisting of an angry and a happy emoticon when emotion had to be reported, or a symbol like male/female picture (see also Fig. 1) if gender had to be reported. The position of the pictures on the task cue corresponded with the position of the buttons that had to be pressed (e.g., if a left button press corresponded with a happy face, the happy emoticon was on the left side of the task cue). This was followed by the target-picture, which appeared together with the task cue (2,000 ms or until a response was given; see Fig. 1). Three practice blocks were administered to ensure the task was understood. The first consisted of 16 trials of the emotion task and the second of 16 trials of the gender task. A block was repeated if the participant failed on more than 25 % of the trials. The third practice block consisted of 40 trials randomly switching between the gender and the emotion task. The three experimental blocks consisted of 72 trials each. One-third of the trials were switch trials (switching from the gender to the emotion task or the other way around), and occurred randomly after two, three, or four repeat trials. The task took about 17 min. Faces were from the Karolinska Directed Emotional Faces Set (Lundqvist et al. 1998). Outcome measures were omission error rate (no button press), commission error rate (an incorrect button press), error rate switch cost (mean error rate on all switch trials minus mean error rates on all repeat trials), and reaction time switch cost (mean reaction time on all switch trials minus mean reaction time on all repeat trials). Please note that all error rates were error percentages. To study the effect of emotional stimuli, we also separately calculated switch costs on gender trials (reaction time and error rate on emotion to gender switch trials minus reaction time and error rate on gender to gender repeat trials), and on emotion trials (reaction time and error rate on gender to emotion switch trials minus reaction time and error rate on emotion to emotion repeat trials).

#### Cognitive Ability

Cognitive functioning was estimated with two subtests of the Dutch Wechsler Intelligence Scale for Children (WISC-III; Kort et al. 2002); Vocabulary, and Block Design. These subtests both have good reliability and correlate highly with Full Scale IQ (FSIQ; Sattler 2001).

#### Diagnostic Material and Questionnaires

The SRS (Constantino et al. 2003) is a valid and reliable quantitative measure of autistic traits (Bolte et al. 2008; Constantino et al. 2003). The ADI-R (Lord et al. 1994) is a comprehensive semi-structured interview for parents or principal caregivers, probing for symptoms of ASD. Scores for each of the three domains (reciprocal social interaction; communication and language; and restricted and repetitive, stereotyped interests and behaviors) reaching specified cut-offs, and evidence of developmental abnormality before the age of 36 months, suggests a DSM-IV (APA 2000) or ICD-10 (WHO 1992) diagnosis of autism (Gray et al. 2008; Rutter et al. 2003). The CBSQ subscales ‘stereotyped behavior’ and ‘fear of changes’ were used to assess these constructs in daily life. This questionnaire also assesses different aspects of social behavior, and consists of 49 items divided in six subscales (Luteijn et al. 1998; de Bildt et al. 2009).

### Procedure

After written informed consent, SRS, and screening questionnaires were obtained from the parents, participants were tested in two sessions. In the first session the ADI-R was administered to parents (in the ASD group), and the WISC-III subtests to the children. In the second session, the gender-emotion switch task was administered to the children, and the CSBQ was filled out by the parents.

As the current study is part of a larger ongoing intervention study, more tasks and questionnaires were administered, but these are not of relevance for the current study. On both sessions the task order was counterbalanced across participants and during the sessions short breaks were inserted. The first session lasted about 70 min for the children and 150 min for the parents. The second session lasted about 90 min for the children and 50 min for the parents.

### Statistical Analysis

First, to determine whether there were differences in switch costs between children with and without ASD, performance on the switch task (error rates and reaction time) was analyzed using repeated measures ANOVAs with trial type as the within subject factor (two levels: switch and repeat trials) and group as the between subject factor (two levels: ASD and TD). Second, as ASD is known to be a heterogeneous group, we expected high variability of performance. Therefore, speed accuracy tradeoffs were studied by ranking groups on reaction times. Within subject influence of reaction time on error rate, and interaction effects of group by reaction time, were investigated using MANOVAs. Third, as cognitive flexibility was expected to be related to stereotyped and repetitive behavior, correlations were calculated between switch costs in error rate, and in reaction time, with the ADI-R repetitive behavior scale, and the CSBQ stereotyped behavior, and fear of changes scales for the ASD group. Finally, to investigate the influence of switch direction (i.e., influence of emotional cues), switch costs on gender trials and emotion trials were compared with a repeated measures ANOVA. These explorative analyses were done for the group as a whole, with group as between subject variable. Both groups were also analyzed separately, to control for the expected high variability of performance within the ASD group.

### Missing Data and Outliers

SRS-scores were missing for one child in the TD group, and CSBQ data missed from two children in the ASD group, because their parents did not complete these questionnaires. There were no significant outliers. All variables were normally distributed except for switch costs in reaction time on gender trials within the ASD group. However, this variable was normally distributed within the whole group.

## Results

### Matching and Assessment

The individual matching was successful as there were no significant group differences in age, estimated FSIQ, and male/female ratio. As expected, the ASD group had significantly higher SRS and CSBQ scores than the TD group (see Table 1).

### Do Children with ASD Show Cognitive Flexibility Deficits?

As expected, all participants performed better on repeat trials than switch trials as they all showed significantly higher commission error rates, *F*(1,60) = 42.81, *p* < 0.001, *partial η²* = 0.42, omission error rates, *F*(1,60) = 17.6, *p* < 0.001, *partial η²* = 0.23, and reaction times, *F*(1,60) = 162.1, *p* < 0.001, *partial η²* = 0.73, on switch trials compared to repeat trials (see Table 2). Although the ASD group had slightly higher error rates, there was neither a significant effect of group on commission error rates, *F*(1,60) = 1.84, and omission error rates, *F* < 1, nor an interaction effect, *p*’s > 0.10 (see Table 2). Furthermore, there were neither significant differences between the two groups on reaction time, *F* < 1, nor a significant interaction effect of group and trial type, *p*’s > 0.10.

**Table 2:** Group comparisons for ASD and TD of commission error rates (CER), omission error rates (OER), and reaction times (RT)

Measure	Group									
	ASD		TD		Switch effect			Interaction		
	Repeat	Switch	Repeat	Switch	*F* (1, 60)	*p*	Partial η²	*F* (1, 60)	*p*	Partial η²
CER	10.9 (5.6)	16.1 (8.3)	9.4 (4.8)	13.4 (7.6)	42.81	<0.001	0.42	0.69	0.41	0.01
OER	3.0 (6.9)	4.6 (8.7)	1.7 (2.0)	3.1 (3.0)	17.60	<0.001	0.23	0.07	0.79	0.00
RT	842.4 (136.5)	955.4 (162.7)	845.0 (131.4)	962.9 (154.7)	162.10	<0.001	0.73	0.08	0.79	0.00

*ASD* autism spectrum disorder, *TD* typically developing

### Is There a Difference Between Children with and Without ASD in Speed Accuracy Tradeoff?

**Fig. 2:**
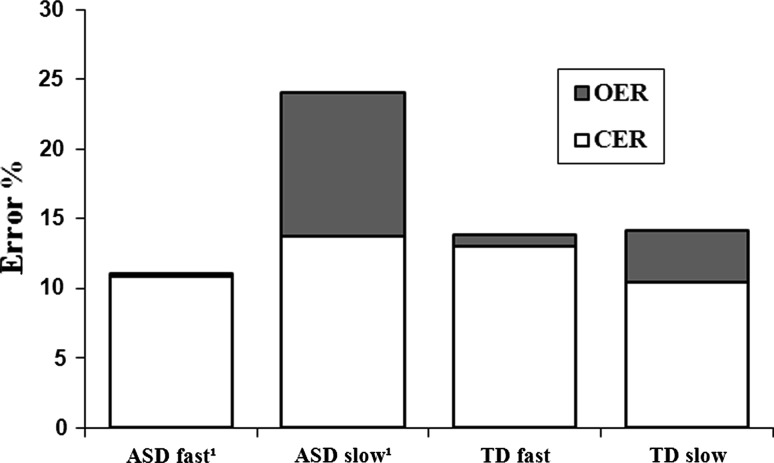
Speed Accuracy tradeoff. *OER* omission error rate, *CER* = commission error rate ¹There was an interaction trend; children with ASD and high reaction times (*n* = 8), had relatively higher omission error rates than TD children with high reaction times (*n* = 8)

In both groups, children with high and low reaction times had similar commission error rates (both ASD and TD, *F* < 1, *ns*), but children with low reaction times had significantly higher omission error rates than children with high reaction times (ASD, *F* (3,27) = 4.03, *p* = 0.02, *partial η²* = 0.31; TD, *F* (3,27) = 4.69, *p* = 0.01, *partial η²* = 0.34; see Fig. 2). Moreover, there is an interaction trend; the children with ASD and high reaction times (*n* = 8), had relatively higher omission error rates than TD children with high reaction times (*n* = 8), *F* (3,54) = 2.21, *p* = 0.10, *partial η²* = 0.11. The reaction times of these relatively slow ASD children (M = 1,037 ms, SD = 126, range 951–1,277) did not approach the maximum possible reaction time (2,000 ms). Hence, the high rate of omission errors is not a result of overall slowness or the inclusion of a too short inter stimulus interval. This ASD subgroup indeed performed relatively poor overall, both in speed and accuracy.

### Is Performance on a Switch Task Related to Stereotyped and Repetitive Behavior?

Within the ASD group, the correlation of the ADI-R repetitive behavior scale with the switch costs in omission error rate, was marginally significant, *r* = 0.34 *p* = 0.06 (see Table 3). The CSBQ stereotyped behavior scale correlated significantly with switch costs in omission error rates, *r* = 0.44 *p* < 0.05, but none of the other correlations were significant (see Table 3 for details).

**Table 3:** correlation within the ASD group (*r*)

Scale	Switch costs		Reaction time
	Error rate		
	Commission	Omission	
ADI-R repetitive behaviour	−0.07	0.34^a^	0.08
CSBQ stereotyped behavior	0.03	0.44*	0.14
CSBQ fear of changes	−0.21	−0.30	−0.11

The correlational pattern was independent of IQ as controlling for IQ did not alter this correlational pattern

^a^This correlation was marginally significant, *p* = 0.06

* <0.05

### Do Children with ASD have Trouble Disengaging from an Emotional Task Set?

**Fig. 3:**
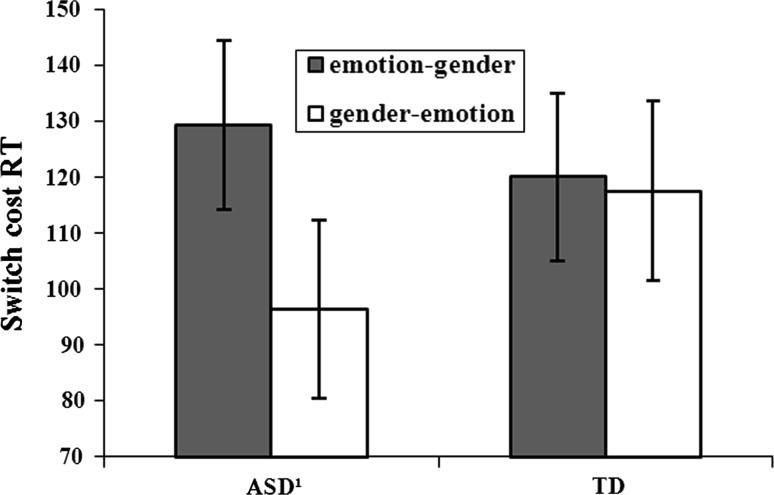
Switch costs in total reaction times on gender and emotion trials. *Error bars* represent standard errors of the mean. *Emotion*-*gender* emotion gender switch trials compared to gender gender repeat trials. *Gender*-*emotion* gender emotion switch trials compared to emotion emotion repeat trials. ¹Switch costs in reaction time were relatively higher on emotion to gender trials than on gender to emotion trials *p* = 0.08

There was no group by switch direction interaction effect in switch costs in reaction time, *F* (1,60) = 2.17, *ns*; commission error rate, *F* (1,60) < 1, *ns*; or omission error rate, *F* (1,60) = 1.04, *ns*. However, given the exploratory nature of this research question, we did run separate follow up analyses for each of the two groups. Within the ASD group we did find a trend for switch direction; the switch costs in reaction time were higher on emotion to gender trials (compared to gender to gender trials) than on gender to emotion trials (compared to emotion to emotion trials), *t* (30) = 1.8, *p* = 0.08. Hence, switch costs were relatively higher when children with ASD had to shift from an emotional to a neutral task set, than when they had to shift their attention from a neutral to an emotional task set. In the TD group, switch costs in both directions were equal, *t* (30) = 0.17, *p* = 0.87. In both groups there were no differences in switch costs in error rates, *p’*s > 0.10 (See Fig. 3).

## Discussion

The goal of the current study was to bridge the gap between cognitive flexibility deficits in ASD as reported in everyday life and the inconsistencies in findings in studies trying to detect these deficits in the laboratory (Geurts et al. 2009b). To this end, performance of children with and without ASD was compared on a switch task with minimal WM-load, and increased ecological validity (as unpredictable switches, and more complex stimuli, i.e., emotional faces, were included). Switch costs on this so called gender emotion switch task, measuring cognitive flexibility, were comparable to switch costs reported in other switch task studies (Poljac et al. 2010; Schmitz et al. 2006; Shafritz et al. 2008; Stoet and López 2010; Yerys et al. 2009). In contrast with our expectation, children with ASD did not show cognitive flexibility deficits on the current task. Nonetheless, in line with the findings of Yerys et al. (2009), an increase in repetitive behavior was related to an increase in switch costs within the ASD group. Apparently, children with ASD that perform poorly (i.e., less accurate) on a switch task also show relatively more repetitive behavior in everyday life. The null findings on the switch task in combination with the observed relation with repetitive behavior, is in correspondence with the finding that there were relatively large individual differences within the ASD group. It appears that only a subgroup of children with ASD show cognitive flexibility deficits, and in the current study, only a subgroup performed relatively slow and was less accurate. Also, children with ASD had higher switch costs in speed of responding, when switching from emotion to gender trials than the other way around.

Our findings are in line with other studies using switch tasks with low WM-load (Schmitz et al. 2006; Stoet and López 2010). However, because of the unpredictable switches (Maes et al. 2010; Stoet and López 2010; Yerys et al. 2009) and increased ecological validity, it was expected that children with ASD would perform worse than children without ASD. There are at least three possible explanations for the current findings.

Firstly, although switches occurred in an unpredictable manner, children did know that switches would occur at some point, so they were still somehow prepared for the switches. In everyday life, children with ASD seem especially rigid when a change of plans or a disruption in their routine happens entirely unexpected. When warned, guided, or prepared for a certain change, children with ASD seem better able to adjust to a new situation (Meadan et al. 2011). A switch task is quite predictable compared to everyday life, and consists of explicit rules. Possibly, children with ASD performed as well as children without ASD because the current task was still too predictable. Moreover, alongside cognitive flexibility, performing a switch task also relies on systemizing skills, as understanding the rules concerning causality, and predictability of outcome is necessary for a good performance on this type of tasks (Lawson et al. 2004). Systemizing skills are thought to be well developed in individuals with ASD (Lawson et al. 2004) and might compensate for the flexibility deficits in children with ASD when performing the current task.

Secondly, a switch task might be an overly pure cognitive flexibility measurement, while in everyday life, cognitive flexibility is never entirely isolated. Cognitive flexibility seems intact in ASD in an artificial isolated form, but it might exacerbate perseverative behavior (Dichter et al. 2010) when combined with other constructs (e.g., WM). Indeed, Schmitz et al. (2006) reported no cognitive flexibility deficits in ASD on a switch task with unpredictable switches and low WM-load, while cognitive flexibility deficits are reported on switch tasks with both unpredictable switches and high WM-load (Maes et al. 2010; Stoet and López 2010). Hence, our choice to reduce the WM-load, to increase the purity of the measurement of cognitive flexibility, might have led to decreased ecological validity.

Thirdly, ecological validity could still be insufficient in the current task for other reasons. Using faces as stimuli probably improves ecological validity, because in everyday life, individuals have to deal with other people’s emotions. However, to prevent that instead of cognitive flexibility, emotion recognition abilities would influence task performance, the current task contained only the most basic emotions. In everyday life individuals with ASD specifically experience problems with recognizing more complex and subtle emotions (Begeer et al. 2008). These subtle emotions might, in particular, require more flexible behavior, as an appropriate response depends more on the context in which the emotion is displayed. It is relatively easier to recognize, and interpret, basic emotions like ‘angry’ and ‘happy’, to predict which behavior is most appropriate, and to act accordingly. Also, the faces are administered on a computer screen and no real social response is needed (Ozonoff 1995). Hence, in future studies the inclusion of more complex emotions might increase the ecological validity (see for other suggestions Kenworthy et al. 2008) to a higher extent.

Some might argue that the task in itself was not the reason for our null-findings, but that the pattern of findings was due to the validity of our ASD sample. We chose not to administer an Autism Diagnostic Observation Schedule (ADOS; Lord et al. 2000) to determine the present ASD characteristics. However, given the thorough diagnostic trajectory all the children in the current sample completed, including a parent report regarding the current ASD characteristics, this not seem to be a plausible explanation for lack of an overall group deficit in cognitive flexibility.

A potentially interesting finding was the trend in the ASD group that slow responding participants were less accurate than fast responding participants, compared to the TD group, where slow and fast responding participants were equally accurate. In both the ASD and TD group, the slower children made more omission errors, but in the ASD group, this contrast was larger, resulting in an interaction trend. The most simple explanation would be that slower children just responded too late more often (i.e., exceeding the time limit). However, this does not seem to be the case as the relatively slower responding children did not show such long reaction times. These relatively large individual differences within the ASD group, with only some participants performing relatively poorly overall (high reaction times as well as high error rates), might indicate that only a subgroup of the ASD population experience pure cognitive flexibility deficits and perform poorly on a switch task. Indeed, the ASD population is known for its variability in both behavior and cognition, and even in the basic features of ASD, i.e., social interaction, communication, and restricted and repetitive behaviors and interest (Happé and Ronald 2008). There are individual differences even in very young children with ASD, in theory of mind, executive functioning, and central coherence, and such individual differences seem also to be present at an slightly older age (Pellicano 2010). In that light, the ASD population cannot be seen as a completely homogeneous group. Probably, only some individuals with ASD perform poorly on switch tasks, and only some show repetitive behavior in everyday life. These large individual differences within the ASD population could explain the high variability in performance within the ASD group in the current study. Especially since seven children with ASD in the current sample did not meet the criteria for repetitive behavior on the ADI-R. It is questionable if the ASD population can be considered and studied as one homogenous group with respect to cognitive flexibility. The high variability within the ASD group makes it hard to find any group differences when comparing the whole ASD group to a TD group.

Additionally, we found that the ASD group showed higher switch costs in reaction time when switching from emotion to gender trials than the other way around. In the TD group switch costs in reaction time were equal in both directions. The current findings are still preliminary, but they do suggest that children with ASD indeed need more time to disengage from an emotional task set (Johnson 2009). Both groups processed emotion trials more slowly than gender trials, but as switch costs were compared, this relatively slow processing of the emotions cannot explain the findings. In the current task, participants had to react to the emotions in the emotion task, and in the gender task, the still visible emotions had to be ignored. A stronger distinction between the gender and emotion task might result in more robust differences between the two groups. Moreover, Johnson (2009) found a similar effect in anxious and worrying individuals. Therefore, it will be important that in a future study frequently occurring comorbid anxiety within the ASD children (White et al. 2009) will be measured to determine whether our pattern of findings can indeed be related to ASD or can be explained by the presence of comorbid anxiety.

In conclusion, children with ASD do not show deficits on the gender emotion switch task, but switch performance is related to the amount of repetitive behavior. Moreover, a subgroup of children with ASD performs relatively poorly overall, and children with ASD seem to have more difficulty disengaging from an emotional task set. The high variability within the ASD group reflects individual differences, and heterogeneity within this population. This implies that instead of focusing on analyses on a group level, an individual differences approach might be more fruitful for future research.
